# GSA-PCA: gene set generation by principal component analysis of the Laplacian matrix of a metabolic network

**DOI:** 10.1186/1471-2105-13-197

**Published:** 2012-08-09

**Authors:** Dan Jacobson, Guy Emerton

**Affiliations:** 1 Institute for Wine Biotechnology, Stellenbosch University, Stellenbosch, 7600, South Africa

## Abstract

**Background:**

Gene Set Analysis (GSA) has proven to be a useful approach to microarray analysis. However, most of the method development for GSA has focused on the statistical tests to be used rather than on the generation of sets that will be tested. Existing methods of set generation are often overly simplistic. The creation of sets from individual pathways (in isolation) is a poor reflection of the complexity of the underlying metabolic network. We have developed a novel approach to set generation via the use of Principal Component Analysis of the Laplacian matrix of a metabolic network. We have analysed a relatively simple data set to show the difference in results between our method and the current state-of-the-art pathway-based sets.

**Results:**

The sets generated with this method are semi-exhaustive and capture much of the topological complexity of the metabolic network. The semi-exhaustive nature of this method has also allowed us to design a hypergeometric enrichment test to determine which genes are likely responsible for set significance. We show that our method finds significant aspects of biology that would be missed (i.e. false negatives) and addresses the false positive rates found with the use of simple pathway-based sets.

**Conclusions:**

The set generation step for GSA is often neglected but is a crucial part of the analysis as it defines the full context for the analysis. As such, set generation methods should be robust and yield as complete a representation of the extant biological knowledge as possible. The method reported here achieves this goal and is demonstrably superior to previous set analysis methods.

## Background

Gene Set Analysis (GSA) has proven to be a useful approach to microarray analysis. The underlying principle of GSA is that aggregate scores are assigned to each Gene Set based on all the individual gene scores within that set. There have been several different methods proposed to assign scores to gene sets [1-8]. Of the approaches published to date, Gene Set Enrichment Analysis (GSEA) [3][7] seems to have become the most commonly used. Of issue though is the fact that GSEA is based on a modified Kolmogorov-Smirnov test. This test can exhibit a lack of sensitivity; is difficult to employ in practical use, and requires at least 1000 permutations to be run. However, it has recently been found [9] that a one-sample Z-test can be very effective with gene sets for detecting shifts from the mean (sets that collectively show up *or* down regulation of their constituent genes). Unfortunately, this will not identify gene sets that have a balance of both up *and* down regulated genes as there will not be the requisite shift from the mean but in statistical terms is rather a change in scale. However, a chi-squared test can be used to good effect to detect such changes in scale and thus find gene sets that exhibit a mixture of up *and* down regulation [9]. Furthermore, Irizarry et al. [9] have shown that the use of a combination of the computationally simple and rapid Z-test and chi-squared methods outperform GSEA. Dinu et al. [10]have extended the Significance Analysis of Microarrays to Gene Set Analysis (SAM-GS). Of further interest is the method described by Efron and Tibshirani [11]which uses a max-mean statistic to target gene sets with only a fraction of the genes differentially expressed and the approach of Falcon and Gentleman [12]which takes into account the fact that overlap exists between different gene sets. A good review of the various statistical approaches has been written by Goeman and Bühlmann [13].

### Gene set generation

Given the discussion above it is clear that considerable effort has been made to apply different statistical methods to GSA, however all of the methods are highly dependent on the very first step: the predefinition of the sets of genes to be analysed. The theoretical combinatorial space for gene sets is quite large and is defined by the binomial distribution of the number of genes in the genome and the size of the desired set:

$$(\begin{array}{l} \text{genes}\\ \text{set}\;\text{size} \end{array}\;)\equiv \frac{\text{genes}!}{\text{set}\;\text{size}!\left(\text{genes}-\text{set}\;\text{size}\right)!}$$

**Figure 1:**
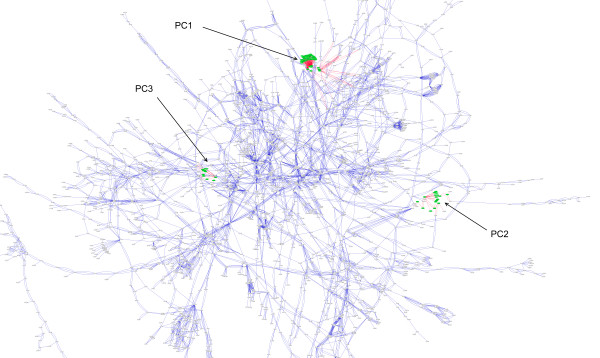
Location of the sets of nodes derived from the first three principal components of the laplacian matrix of the metabolic network, topographically depicted on the metabolic network itself.

Thus, if one wanted to create all of the possible unique sets with 8 members for the ~6000 genes present in the yeast genome, there would be (6000 choose 8) = 4.1 × 10^25^ sets. This is clearly an unfeasible number of sets to generate, much less evaluate. Instead, methods to date have used extant biological knowledge to generate relatively small numbers of sets to be evaluated. One of the common approaches taken for set generation is to simply place the genes involved in a specific pathway into a set. This approach suffers from the fact that pathways are merely human abstractions that are useful for visualisation and interpretation, as they can serve as mnemonic devices for areas of metabolism. However, in isolation, single pathway sets do not reflect the continuously connected nature of biological networks. The metabolic network of *Saccharomyces cerevisiae* (and the location of some of the gene sets found by PCA) can be seen in Figure 1. It is clear that this is a complex, interconnected network and, as such, any attempt to use simple pathway representations of it will inevitably be an incomplete representation of the underlying network. We therefore propose that many “pathway sets” are, by definition, rather arbitrary and incomplete, and gene expression patterns may therefore be potentially missed due to improper/incomplete set generation. We suggest that a method that semi-exhaustively partitions the network into overlapping sets would be a better approach to set generation. In order to achieve this we have devised two algorithms that use the Principal Component Analysis of a Laplacian matrix of a metabolic network to do gene set generation. We have also devised a hypergeomtric test to determine which of the genes in the sets identified by gene set analysis are likely to be driving set selection. We have used the resulting gene sets to analyse a publicly available microarray dataset and compare the results obtained from our algorithms (and their respective parameters) to each other as well as to results obtained with traditional pathway sets.

It is important to note that our intent in this paper is not to compare GSA methods to standard parametric statistical approaches (such as t-tests) as there is ample literature on GSA to show its usefulness in difficult datasets (in which there are many orthogonal factors at play which can make a dataset difficult to analyse with other approaches). Rather, our intent is to select a relatively simple dataset with which to make the point that the existing state-of-the-art GSA methods that use isolated pathways are very prone to miss significant amounts of the signal (as they are not capturing the entire metabolic set space) and to report insignificant genes as they are simply associated with a set that is deemed to be collectively significant. As such, both the false negative and false positive (due to “passenger” genes) rates of GSA are high when using isolated pathways. We believe that we have shown that we can address these issues with our method. To demonstrate this we have chosen a straightforward, publicly available dataset with a simple perturbation for which this can easily be demonstrated. We have used this dataset to simply highlight the differences in the results generated by our method rather than to do a full-blown biological interpretation of the microarray results.

## Methods

### Affymetrix probeset to yeast genome mapping

The sequences for each of the individual probes of the Affymetrix Yeast 2.0 Genechip were mapped to the Yeast Genome by the use of blastn [14]. A Perl program was written to perform the following tasks: 1) extract 100% identity matches (over the full length of the probe) from the blastn results; 2) assemble the probes into probesets and 3.) model the resultant probeset-to-gene relationships as an Affymetrix-probeset-to-Genome graph.

### Single pathway set creation

In order to compare our method to how GSA of pathways has been done in the past it was necessary to create single pathway-specific sets. A Perl program was written to parse the XML files downloaded on June 27, 2011 from KEGG [15] and the genes listed in each pathway file were used to create a simple set for each pathway. This is analogous to how pathway sets have been created for GSA previously.

### Metabolic network and Laplacian matrix creation

A Perl program was written to parse the XML files downloaded on June 27, 2011 from KEGG [15]. Nodes were created for compounds, reactions and genes, and edges created between genes and the reactions they are involved in as well as between compounds and the reactions they are substrates for or products of. The result of this process can be seen in Figure 1 as visualised by Cytoscape [16]. The resulting metabolic network was used for all subsequent set generation. A reference structure within the Perl program which reflected the nodes and edges in the graph was used to identify adjacency and degree parameters for each node and thus generate the corresponding Laplacian matrix.

### Principal component analysis

Principal component analysis of the Laplacian matrix was done in R with the prcomp function. Qlucore v2.2 (Lund, Sweden) was also used for PCA model creation and visualisation of principle components during the exploratory phase of algorithm development.

### Set theoretic analysis

Set theoretic analysis (intersects, differences, and sizes) of the gene sets and pathway results sets was done in Perl with the use of the Set::Scalar library v 1.25 [17].

### Graph theoretic analysis

Graph theoretic analysis was done in Perl with the use of the Graph library v. 0.94 [18].

### Threshold-based set creation algorithm

As became clear from examining the PCA score plots, as well as plots of scores across all components, a gene or group of genes may have different scores in different principal components. As such, we decided to create sets at a number of different thresholds to investigate whether this approach would give more or less sensitivity in gene set analysis. Thus for each principal component the positive scores were compared against a series of integer thresholds (1 through 10) and if the score at a principal component was greater than the threshold it was added to a set created for that principal component. A similar procedure was followed for the negative scores at each principal component with the score required to be less than the negation of the integer threshold. Genes in these sets were then mapped to Affymetrix probeset ids with the use of the aforementioned Affymetrix-probeset-to-Genome graph (described elsewhere in the Methods section), and the matched probes substituted for the genes in the set. Sets that contained more than, or equal to, five probeset ids were kept for further analysis. The resulting sets were subsequently printed out in the .gmt set format used by Efron and Tibshiran (2007) [11]. This algorithm was implemented in Perl.

### Step-function-based set creation algorithm

In a separate effort to determine the effects of groups of genes clustering at distinct score ranges within each component on gene set creation and performance an algorithm for set creation was created as follows. An initial empty score-range set was created as an array and held in memory. Scores at each component were sorted in numerical rank order. For positive scores greater than one, neighbouring rank order scores were subtracted from one another and if the difference was < 1 they were added to the existing score-range set, if the difference was > 1 the a existing score-range set was closed and a new one (for the next score range) was created and the new gene added to it. This process was repeated across all of the scores in each principle component. A similar procedure was used for the negative scores less than negative one. This algorithm was implemented in Perl.

### Gene set analysis with newly generated sets

We used the sets created by the Laplacian PCA method described above for the max-mean method by Efron and Tibshiran (2007) [11]. In order to test the new sets on a data set that would likely have a limited number of subtle changes on metabolism, we selected a data set that examined the effect of an O-glycosylation inhibitor, OGT2468, on gene expression deposited by Javier Arroyo. They used the *Saccharomyces cerevisiae* strain SEY6210 and analysed the global transcriptome in the absence of the OGT2468 (but with the corresponding amount of DMSO, 0.1%) and in the presence of 0.1 μM of OGT2468. They report that “yeast cells exposed to OGT2468 in YPD growth medium show a significant inhibition of mating, filamentation and induction of cell wall compensatory mechanism.” The resulting microarray data was downloaded from the Gene Expression Omnibus (series id GSE12193) [19]. Specifically, the microarrays used were those for the DMSO control (GSM306567, GSM306565, GSM306569) vs. the cells treated with 0.1 μM of OGT2468 (GSM306573, GSM306577, GSM306581). The microarray data was normalised in R with the RMA method [20] and the resulting log2 transformed data used for GSA. GSA was performed on this data with the following settings: resp. type = "Two class unpaired", nperms = 1000, minsize = 2 and FDR cut = 0.05. The GSA analysis for the largest number of sets (3481 Threshold 1 sets) ran in 2.3 minutes on a single CPU Intel Xeon E5620 2.40 GHz CPU. The genes found in the sets created at PCA score thresholds 1 through 10 which were determined to be differentially expressed by GSA were tested with the hypergeometric test described above. The genes selected by this test were then used to create genes-only and compounds-containing pathway-centric graphs as described below. These graphs were visualised in cytoscape. Fold change values were calculated as the ratio of the average expression values for the samples containing 0.1 μM of OGT2468 to the DMSO controls, and used as node attributes to colour the gene nodes in cytoscape. The negative reciprocal was taken of ratios less than 1.

### Hypergeometric enrichment test to determine genes most responsible for set selection

In order to determine which genes identified by GSA on our newly derived sets are drivers we implemented a hypergeometric test. The probability (p) of obtaining any such set of values is given by the hypergeometric distribution:

$$p=\frac{\left(\begin{array}{c} a+b\\ a \end{array}\right)\left(\begin{array}{c} c+d\\ c \end{array}\right)}{\left(\begin{array}{c} n\\ a+c \end{array}\right)}$$

Where $\left(\begin{array}{c} n\\ k \end{array}\right)$ is the binomial distribution, *a* = Number of times the gene is found in significant sets; *b* = Number of times all other genes are found in significant sets; *c* = Number of times the gene is found in non-significant sets; *d* = Number of times all other genes are found in non-significant sets, and *n* = a + b + c + d. A Perl program was written to parse each of the gene set analysis results as well as the original sets used for the analysis after thresholding, and from these two sources calculate a, b, c, d and n. The two tailed Fisher module of the Text::NSP Perl package [21] was used to test for significance using these values and multiple hypothesis testing corrected for with the Holm-Bonferroni method [22]. Genes with a q-value less than or equal to 0.1 were reported as significantly enriched (i.e. drivers) and included in a pathway-centric network reconstruction for visualisation and interpretation.

### Pathway-centric network reconstruction for visualisation and interpretation

As was mentioned in the introduction, pathways are really human abstractions of subgraphs of a metabolic network that are particularly useful as mnemonic devices for contextual visualisation. Unfortunately, visualizing the network with all of the reaction, compound gene and pathway nodes present is overwhelming and as such difficult to interpret. If a biologist can see that the genes selected are part of a well-known pathway it helps them to interpret what part of the metabolic network they are examining. By evaluating the results in this linked metabolic context, one is able to see relationships between areas of metabolism that simply would not be apparent by looking at lists of genes or lists of pathways. Thus, we have created two types of visualisations in order to better show the metabolic context of the results. The first visualisation just contains the significant genes and the pathways that they are associated with. This is a useful and relatively simple way to visualize the results. However, there are cases where related pathways are affected but do not show up as connected without the compounds being included in the network. Inclusion of all of the compounds leads to a very complex figure so we have chosen to simply create a single edge between pathways if they share one or more compounds.

**Figure 2:**
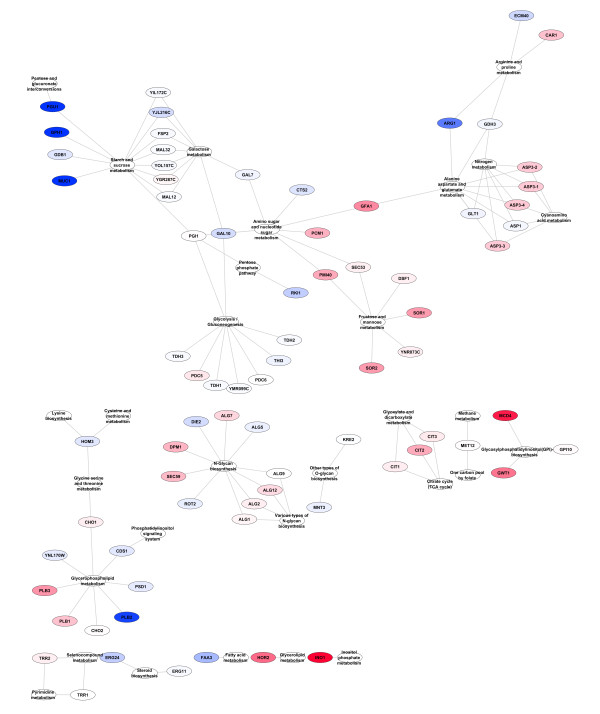
**Genes from PCA scores threshold = 1 derived sets found to be significantly differentially expressed in their pathway-centric context.** Nodes are coloured different intensities of blue (decrease) or red (increase) based on the fold change between treatment and control.

Raw KEGG XML files actually pre-group all genes, reactions and compounds into these contextualised pathways. In order to provide these sorts of visualisation cues a pathway-centric view of the metabolic network was therefore constructed as follows:

A Perl program was written to parse the KEGG XML files such that a node was created for each pathway as defined in KEGG. An edge was created between the pathway node and each gene or compound that is associated with that pathway. A gene determined to be significant by GSA and the subsequent hypergeometric test was used as a seed for a breadth first search of the pathway-centric graph with a radius of one. This was done iteratively for each gene and the union taken of the resulting subgraphs (for examples of outputs see Figure 2). A similar procedure was followed for the compound-linked-pathways view with the additional step of the creation of a single edge between pathways that shared one or more compound.

#### Genes-only and compounds-containing graphs

Two different graphs were then created, one with and one without compounds. For the genes-only graph, compounds and their associated edges were simply removed from the graph. The compounds-containing graph was constructed by removing all compounds with a degree less than two, such that compounds only served to link pathway nodes together. The genes-only view of the graph is easier to visualise and interpret whereas the graph containing pathway-linking compounds was useful in showing the connectedness (or lack thereof) of the subgraphs containing differentially expressed genes.

### Gene Ontology and Funcat enrichment analysis

Genes that were found by the threshold 1 method but not by the pathway GSA were checked for GO Enrichment by GOEAST [23] and FunCat Enrichment at MIPS [24].

## Results and discussion

### Graph representation of a metabolic network

Mathematically, a metabolic network can be represented as a graph, G = (V,E), where V is a set of *n* nodes and E a set of *e* edges (connections) between nodes. Let A(G) = A be the adjacency matrix of G such that each element A_ij_ is assigned a value of one if the corresponding nodes are adjacent and zero if they are not. The graph can be further described by a Laplacian transform of the adjacency matrix. The Laplacian matrix L(l_i,j_)_nxn_is defined as:

$$l_{\text{i,j}}\mathrm{:=}\left\{\begin{array}{l} \text{deg}(v(i))\text{if}\;i=j\\ -l\;\text{if}\;i\neq j\;\text{and}\;v\left(i\right)\text{is}\;\text{adjacent}\;to\;v(j)\\ 0\;\text{otherwise} \end{array}\right)$$

where deg(v(i)) denotes the degree of v(i), i.e. the number of edges incident to (i). Thus, the Laplacian Matrix is the difference between the diagonal Degree Matrix (D) and the Adjacency Matrix (A).

L = D - A

A number of graph theoretic properties of a graph can be derived from its Laplacian matrix and the eigenvector and eigenvalues thereof, including the number of connected components in the graph; its algebraic connectivity (Fielder value); its spectral gap, etc. In fact, the PCA of a graph and spectral graph clustering have been linked previously by Saerens et al. [25].

### Principle component analysis

Given a matrix, one can use multivariate statistical methods such a Principal Component Analysis (PCA) to try and find correlative relationships amongst the vectors. PCA is a bilinear modelling method which gives a visually interpretable overview of the most salient information in large, multidimensional datasets. By plotting the principal components it is possible to view statistical relationships between different variables in complex datasets and detect and interpret object groupings, similarities or differences, as well as the relationships between the different variables [26]. As described in the Methods section a graph was created from the KEGG database [15] and a Laplacian matrix derived from said graph. The Laplacian matrix produced, while not a typical object-variable data matrix, may still be analysed with multivariate methods. It was hypothesised that a principal component model would enumerate groups of nodes within the graph with similar topological structure, with the thought that similar columns within the Laplacian matrix would explain a certain amount of ‘variance’ in the matrix. Accordingly, we then performed PCA on the Laplacian matrix with the hopes of finding an exhaustive set of structures within the graph that could be used for gene set generation.

### Gene set generation with score thresholds and step-functions

It was observed that the scores for each principal component often generated discontinuous clusters of objects. Two algorithms for set generation were therefore developed: one based on a step function that took the score discontinuities into account; and another that specified several predetermined thresholds. The sensitivity levels of both these methods were subsequently compared. These algorithms were used to generate gene sets from the genes found in each of the 2656 principal components (see Methods).

### Gene set analysis with new gene sets

We used the sets created by the Laplacian PCA method described in the Methods section to perform the max-mean method of GSA [11]. In order to test the new sets on a dataset that would likely have a limited number of subtle changes on metabolism, we selected a data set that examined the effect of an O-glycosylation inhibitor. The analysis was done as described in the Methods section and the results described below.

### Hypergeometric enrichment test to determine genes most responsible for set selection

**Figure 3:**
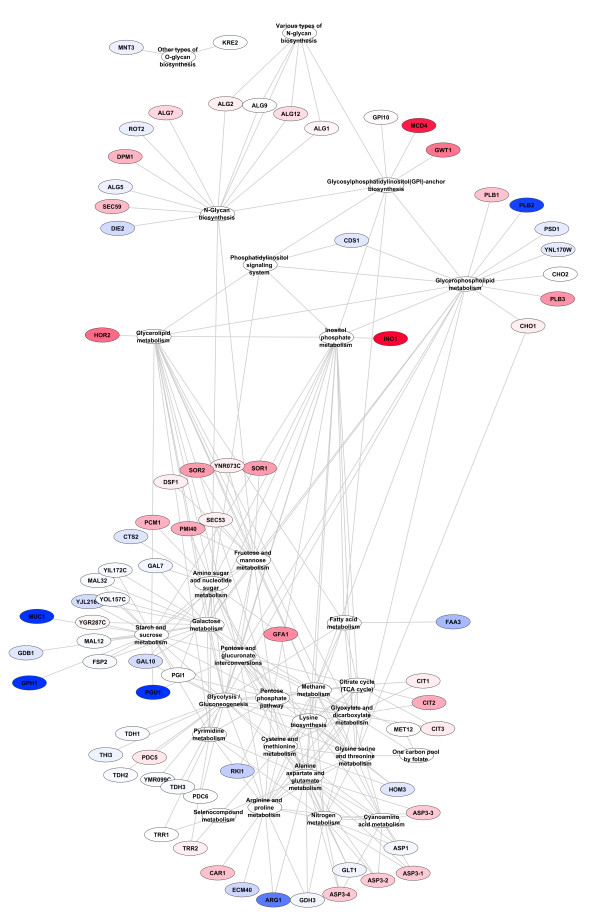
**Genes from PCA scores threshold = 1 derived sets found to be significantly differentially expressed in their pathway-centric network reconstruction including edges between pathways that share at least one compound.** Node colouring as described in Figure 2.

**Figure 4:**
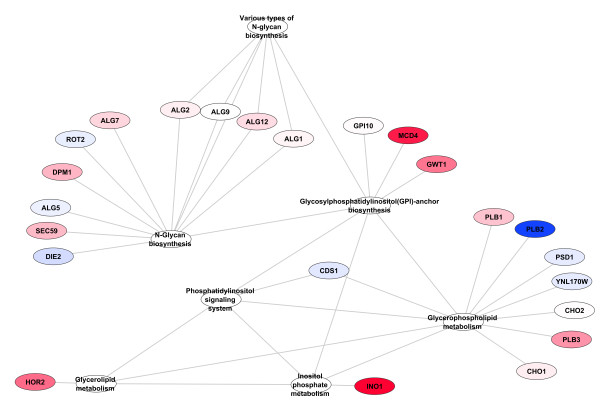
**Zoom in of the Genes and Pathways from Figure**3**that are involved in Glycerolipid metabolism, Glycerophospholipid metabolism, Glycosylphosphatidylinositol(GPI)-anchor biosynthesis, Inositol phosphate metabolism, N-Glycan biosynthesis, and the Phosphatidylinositol signaling system.** Node colouring as described in Figure 2.

One of the difficulties faced in Gene Set Analysis is that it is unclear which of the genes within a set found to be significantly and collectively different are most responsible for that difference. This means that each significant gene set likely has a subset of genes that really account for the difference that is detected (“drivers”); and a separate subset of genes that do not significantly contribute to the greater set’s difference (“passengers”). Fortunately, due to the semi-exhaustive nature of our set creation algorithm we have the ability to test for the likelihood of set members being drivers or passengers. If a gene is found in a number of significant sets at a considerably higher frequency than one would expect to see at random then it is more likely that the gene in question is a driver. In order to determine which genes identified by GSA using our newly derived sets are drivers we implemented a hypergeometric test as described in the Methods section. Those genes considered to be drivers were included in a pathway-centric network reconstruction for visualisation and interpretation (see Methods) as seen in Figures 2, 3, 4.

### High numbers of components are required to model a metabolic laplacian matrix

First, we noticed upon examining the Laplacian PCA model that it needed 2655 principal components to explain all of the variance in the matrix (the same number of total ‘variables’ in the matrix). This is unusual as PCA models are normally quite efficient at reducing the dimensionality of a data set. In this case we believe that it suggests that the model is likely semi-exhaustively explaining local structures in the graph. Additional file 1: Figure S1 is a plot of the percentage of variance explained by each principle component. It is easy to see from this plot that the variance being modelled is spread out quite broadly over the 2655 components with almost all of the components individually explaining less than 0.08% of the variance.

### Individual components model local areas of the graph

In order to test this hypothesis visually we extracted nodes from each principle component and examined their locations in the metabolic network. Figure 1 is an overview of the metabolic network with the significant nodes found in the first three principal components (according to an imposed score threshold of 1), identified in green and their adjacent edges identified in red. It is clear that the first three principal components are identifying distinct, localised structures in the graph. With the hypothesis that genes that are closely related to one another in the metabolic network are likely to be co-regulated we believe that each principal component in the model is a candidate for one or more sets of genes to be tested by GSA. To examine this further we looked at the graph structures being located by many of the principal components. We found that the principal components are finding graph structures with highly connected sets of genes that will likely be good candidates for GSA. We continued this analysis through many higher components of the model to confirm that this was indeed occurring throughout a broad range of components. To confirm this observation the distance between all genes in each set was determined and the average distance within each set calculated to be 8.4. Given that there is a distance of one between a gene and the reaction it is associated with and a distance of two (reaction to compound to next reaction) between reactions, this means that in each set, on average, each gene is being associated with genes involved the first, second or third neighboring reactions. The distances between all genes in the metabolic network have been calculated and their sorted distribution is shown in Additional file 2: Figure S2. The red arrow indicates the average intra-set gene distance. As such, the sets contain only about 20% of the possible gene pair distances. Thus, it appears that sets are modelling relatively local topologies in the network.

### Semi-exhaustive nature of PCA-Graph gene set creation

We examined the distribution of PCA scores for each gene in the model across all of the principal components. Almost all genes participate in multiple principal components. This indicates that sets generated from the principal components from the PCA of the Metabolic Laplacian matrix will be topologically exhaustive, that is to say covering the combinatorial space as constrained by the graph in an overlapping fashion. To confirm this we examined the number of times that each gene was found in sets created by a principal component score threshold of one. Additional file 3: Figure S3 shows the rank order distribution of the number of sets each gene is a member of. Only two genes belong to only one set and some genes belong to as many as 336 different sets. As such, it is clear that in GSA almost every gene would be tested in combination with many other groups of genes, which gives our method a higher likelihood of finding co-regulated sets of genes. We checked the location of the 20 genes that belong to the fewest gene sets and found that they typically are either located in the extreme leaf nodes of the large network or in the small disconnected subgraphs. Intuitively, this makes sense as members of the outer extremities of the large network and the disconnected subgraphs will be part of fewer graph structure variants and therefore occur in fewer principal components, and thus belong to fewer sets derived from the principal component scores.

#### Degree of overlap amongst sets

In order to determine the level of overlap between the sets generated by this approach an all-against-all comparison of the sets was done by way of set theoretic intersects. For the 3481 sets generated at a score threshold of one, an all-against-all comparison is comprised of 10,753,203 set intersects. The number of sets intersecting with each individual set was calculated and plotted (Additional file 4: Figure S4A). As can be seen from Additional file 4: Figure S4A many of the sets do have intersects with one another ranging from as few as 2 to as many as 2702. In order to achieve a semi-exhaustive coverage of the graph’s local topology this sort of overlap is desirable, as long as the degree of overlap is not so high that the sets become effectively redundant. In order to determine the degree of the overlap amongst sets the size of each intersection was calculated, followed by the number of set intersections of each respective size (Additional file 4: Figure S4B). Of the 10,753,203 set intersects performed, 8,225,500 showed no shared genes at all, 1,125,959 shared one gene, and 519,333 shared 2 genes. As can be seen in Additional file 4: Figure S4B the number of set intersections with higher degrees of overlap drops very quickly. This would appear to be a very desirable result as it appears that the Laplacian PCA is yielding sets that thoroughly cover local topological structures in the graph without introducing an excessive level of overlap, i.e. redundancy, in the sets. As such the set intersection space is actually quite sparse as 80% of the potential set intersections show no overlap at all. This again emphasizes that local topological structures in the graph are being modelled by PCA, as one would not expect there to be overlaps of subgraphs (and the sets generated from them) that are topologically separated from one another.

#### Set size

The sizes of the sets vary at each principal component depending both on the region of the graph being modelled by that component and the magnitude of the score threshold employed. Additional file 5: Figure S5 shows the distribution of set sizes when a score threshold of +/− 1, 5 or 10 is used. The set sizes range from 5 to 92 members with an average set size of 27 genes.

#### Hypergeometric test

The hypergeometric test resulted in a ten-fold reduction in the number of genes from the threshold 1 sets up to a forty-nine-fold reduction from the threshold 5 sets, thus simplifying the resulting network that needed to be visualised and analysed.

### Results from sets derived from different PCA score thresholds

Sets created at different PCA thresholds naturally have different set sizes and composition and as such may have slightly different sensitivities in finding differential changes in some portions of the metabolic network. The threshold-based set generation algorithm produced 3481, 2703, 1487, 745, 331, 168, 80, 49 and 35 sets for threshold 1, 2, 3, 4, 5, 6, 7, 8, 9, and 10 respectively for a total of 11,216 sets.

In order to evaluate the efficacy of sets created at different thresholds, a set theoretic approach was used to determine the effects of these thresholds employed during set generation on the pathways identified by GSA. Lists (sets) of pathways with significant differences were generated from the results of GSA performed with sets generated with PCA score thresholds 1 through 7 (see Methods). Thresholds 6 through 10 yielded no results after the application of the hypergeometric test. This is likely due to the relatively small number of genes and sets generated at the higher threshold levels. As such, there are insufficient differences in global and individual set frequencies for the hypergeometric test to discriminate between driver and passenger genes (i.e. to generate sufficiently low p-values). The set theoretic difference was then determined between the pathway sets resulting from PCA scores thresholds 2 through 5 as compared to the pathway set found using a PCA scores threshold of 1.

All but one of the pathways found at thresholds 2 through 5 were also found with a threshold of 1, the one exception being one gene in Aminoacyl-tRNA biosynthesis found at threshold 3. As expected, the threshold 1 results contained many pathways not found at the other thresholds, specifically: 11, 17, 22 and 24 more pathways were found with threshold 1 than with thresholds 2, 3, 4 or 5 respectively. However, as the different thresholds contain subsets of the pathways and genes found in threshold 1 they can be used as a ‘zoom-in, zoom-out’ method to view focused portions of the differentially changed network. Figure 2 contains the results of sets generated with a PCA scores threshold of 1. Figure 3 is a slightly different view of the results, containing edges between pathways that share one or more compounds. Although somewhat more complex, it clearly shows the linkages between genes in the result sets. Of note is the connections between the pathways seen at the top of the figure that show the connections between N-Glycan biosynthesis, the Phosphatidylinositol signaling system, Inositol phosphate metabolism, Glycerophospholipid metabolism, Glycerolipid metabolism and Glycosylphosphatidylinositol(GPI)-anchor biosynthesis (all but one of which were missed by the single-pathway method) which can be seen in more detail in Figure 4.

### Comparison of threshold-based sets to step-function-based sets

The step-function-based algorithm generated 1704 gene sets. The lower number of sets generated is likely due to the fact that the step-function identified many groupings with less than five members which did not qualify as a set for GSA purposes. The GSA results from sets generated by the use of a step-function on PCA scores was compared to the results generated at a score threshold of 1. Similar to the threshold comparisons the step-function identified three pathways (Cysteine & methionine metabolism, TCA cycle and Methane metabolism) not found by the threshold 1 method. The threshold 1 method also found 18 pathways that were not found by the step-function method. This likely means that the entire positive or negative branch of a principle component is accurately modelling a topological structure and that subgraphs within that topology are not generally needed to increase the sensitivity of gene sets to be used for GSA.

### Comparison of threshold 1 sets to single pathway sets

**Figure 5:**
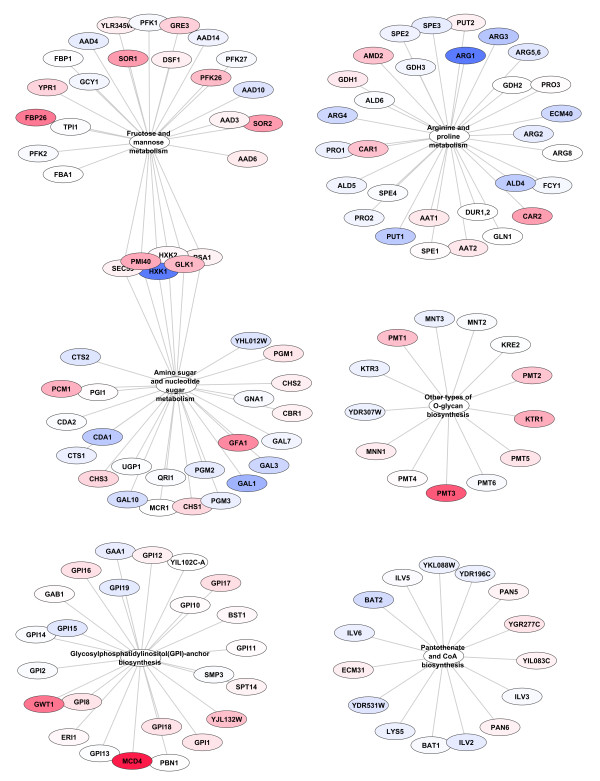
Results from single pathway sets.

As has been discussed above, threshold 1 sets, with very few exceptions, give the most complete view of differential changes occurring in the metabolic network. In order to compare this new set generation method to previous approaches we created single pathway sets (see Methods) which are the type of sets that have traditionally been used in the past. We ran GSA on the 69 single pathway sets described above and compared the results to those generated by the use of threshold 1 sets. The results of the single pathway results can be seen in Figure 5. The single pathway sets only identified 6 pathways as opposed to the 30 identified by the threshold 1 method. Additionally, because there is comparatively little overlap in the single pathway sets it is not possible to use a hypergeometric test to determine which of the genes are drivers of the set statistic and which are simply passengers. Furthermore, with single pathway sets it is much harder to determine how the sets are related to each other within the metabolic network. It is clear that the results from Threshold 1 as presented in Figures 2, 3, 4 are much more comprehensive that those from single pathway sets as presented in Figure 5.

Single pathway sets found 20 (true positives) of the 76 genes found to be differentially expressed by the Threshold 1 method, thus it missed 56 genes (false negatives) found by the Threshold 1 method. Furthermore, it included 111 apparent passenger genes (false positives) that were present presumably because the pathway set containing them was found to be significant and not because they contributed to the signal.

The results of the threshold 1 analysis could certainly be presented as lists of genes or as lists of different pathways. However, we are attempting to point out the limitations of precisely this isolated pathway mode of thinking, the effects of which have been noted elsewhere in the literature:

“The classical method of metabolic engineering, identifying a rate-determining step in a pathway and alleviating the bottleneck by enzyme overexpression, has motivated much research but has enjoyed only limited practical success. Intervention of other limiting steps, of counter-balancing regulation, and of unknown coupled pathways often confounds this direct approach [27].”

Thus, it would appear that there have been many attempts at metabolic engineering in which researchers have not taken into account the fact that pathways don’t exist in isolation but are rather all interconnected. As such, many attempts at metabolic engineering fail as they are based on an overly simplistic model. Therefore, we contend that seeing the genes in the context of the metabolic network is a more accurate way to portray and understand what is really occurring in metabolism. Pathway names are very useful mnemonic devices to remind one what area of metabolism is involved, but should not be used to artificially isolate gene functions from one another.

Our method finds differentially expressed genes involved in N-Glycan biosynthesis, the Phosphatidylinositol signalling system, Inositol phosphate metabolism, Glycerophospholipid metabolism and Glycerolipid metabolism that were missed by the single-pathway method. Furthermore, it provides the visual context for how these pathways are interlinked and how they are linked to Glycosylphosphatidylinositol(GPI)-anchor biosynthesis (the one related pathway which the single pathway method does find). These findings and the relationship between them would seem to be an important aspect of the biology that was missed by the single-pathway approach.

In addition, in order to determine the central biological themes that our method found but were overlooked by the single pathway GSA method, all of the genes that were found by our method and not found with single pathway GSA were subjected to GO Enrichment analysis and FunCat Enrichment analysis as described in the Methods section. Although it is beyond the scope of this paper to do a full biological interpretation of these genes, we have discussed them briefly below and included the full GO and FunCat Enrichment results as Supplementary Material.

#### FunCat enrichment

The general categories of the functions that our method finds that would have been missed previously include, as identified by FunCat enrichment: amino acid metabolism, nitrogen, sulphur and selenium metabolism, carbohydrate metabolism, lipid, fatty acid and isoprenoid metabolism, secondary metabolism, glycolysis and gluconeogenesis, tricarboxylic-acid pathway (citrate cycle, Krebs cycle, TCA cycle), metabolism of energy reserves (e.g. glycogen, trehalose), complex cofactor/co-substrate/vitamin binding and protein modification (N-glycosylation) [Additional file 6].

#### Gene Ontology enrichment

Simply looking at the GO Enrichment diagrams gives one a good sense of how much pertinent biology was found with our method that was missed by pathway GSA. It is clear that we are finding core biological themes in N-linked protein glycosylation, which itself is interesting given that it was O-linked glycosylation that was inhibited. In addition, lipid, phospholipid and glycerophospholipid biosynthesis are affected which links nicely with the enrichment for GPI anchor biosynthesis. There are further indications that branched chain and aromatic acid metabolism is affected as well as the TCA cycle and redox metabolism. Furthermore, it is clear that there is an effect on a number of genes involved in the cell wall, ER and plasma membranes, as one would expect to see with the inhibition of protein glycosylation, which would be likely to affect a number of integral membrane proteins [Additional file 7, Additional file 8 and Additional file 9].

#### Differentially expressed genes

A list of the differentially expressed genes found by our method, including their systematic and gene names as well as their descriptions have been included as supplementary material [Additional file 10].

#### Gene sets and software availability

The gene sets (affy probeset ids) generated by threshold 1 are included as supplementary material [Additional file 11]. Software used for the analysis is available upon request to the first author.

## Conclusions

To our knowledge there is no report in the literature of the use of Principal Component Analysis of the Laplacian matrix of a metabolic graph for any purpose, including set generation for Gene Set Analysis. As such, it appears that this is a novel method with which to find local topological structures in metabolic networks. We have shown that sets generated from the metabolic networks are semi-exhaustive in that there are many partial set overlaps, but the degree of overlap is relatively low. The fact that each gene is a member of many sets allowed us to devise a hypergeometric enrichment test to determine which genes were likely to be driving the set statistic and which were likely to simply be passengers, and could thus be pruned away from the results set. We have further shown that the structure represented by each signed half of each principal component (greater than or equal to a score threshold of 1) is adequate for set generation. Further stratification of each principal component, whether by threshold or step-function methods did not significantly increase sensitivity. However, the thresholding method did prove to be useful as a ‘zoom-in, zoom-out’ function for biological interpretation of the results. When compared to traditional pathway sets this method appears to be much more sensitive as it is a better representation of the underlying complexity of a metabolic network. Furthermore, the method applied here allows one to see the full context of the genes likely to be driving the set statistics rather than as simply lists of pathways each containing an unknown number of driver and passenger genes.

## Competing interests

The authors declare that they have no competing interests.

## Authors' contributions

DJ conceived of the study, created the graph and Laplacean matrix used in this paper, wrote the R code to perform the PCA and the Perl code for set generation, set analysis, and hypergeometric testing, wrote the R script to perform the GSA analysis, did the subsequent interpretation of both the PCA and GSA results, created the figures and wrote the manuscript. GE performed the initial PCA of a Laplacian matrix in Matlab and provided the PCA scores that DJ used to develop the initial prototype, participated in discussions about the potential applications of this approach and edited the manuscript. All authors read and approved the final manuscript.

## Supplementary Material

Additional file 1: Figure S1 Percentage Variance Explained by each principal component. Click here for file

Additional file 2: Figure S2 Gene to Gene Distance Distribution. Click here for file

Additional file 3: Figure S3 Rank order distribution of the number of sets that each gene is contained in. Click here for file

Additional file 4: Figure S4 A) number of other gene sets that intersect with each gene set. B) Number of occurrences of gene overlaps between sets as found by set intersections. Click here for file

Additional file 5: Figure S5 Set sizes generated at each positive and negative arm of each principal component at thresholds 1, 5 and 10. Click here for file

Additional file 6 FunCat Enrichment of the False Negatives from traditional GSA pathway sets. Click here for file

Additional file 7 GO Enrichment (Biological Process) of the False Negatives from traditional GSA pathway sets. Click here for file

Additional file 8 GO Enrichment (Molecular Function) of the False Negatives from traditional GSA pathway sets. Click here for file

Additional file 9 GO Enrichment (Cellular Location) of the False Negatives from traditional GSA pathway sets. Click here for file

Additional file 10 Gene ID, Name and Description of metabolic genes found to be differentially expressed by GSA-PCA. Click here for file

Additional file 11 The Gene Sets Generated with PCA Score Threshold 1. Click here for file
