# Supplementary figures and images for: GSA-PCA: gene set generation by principal component analysis of the Laplacian matrix of a metabolic network
Source: BMC Bioinformatics. 2012 Aug 9;13:197. doi: 10.1186/1471-2105-13-197 (PMC3626710; doi:10.1186/1471-2105-13-197)

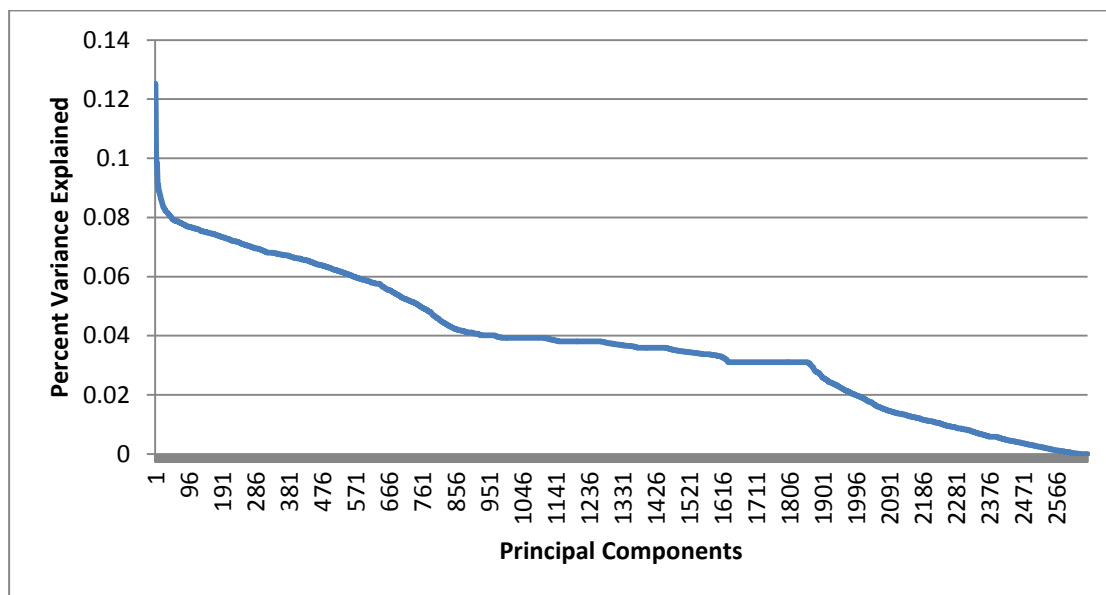

Supplement: Additional file 1: Figure S1 — Percentage Variance Explained by each principal component. [file 1471-2105-13-197-S1.pdf]

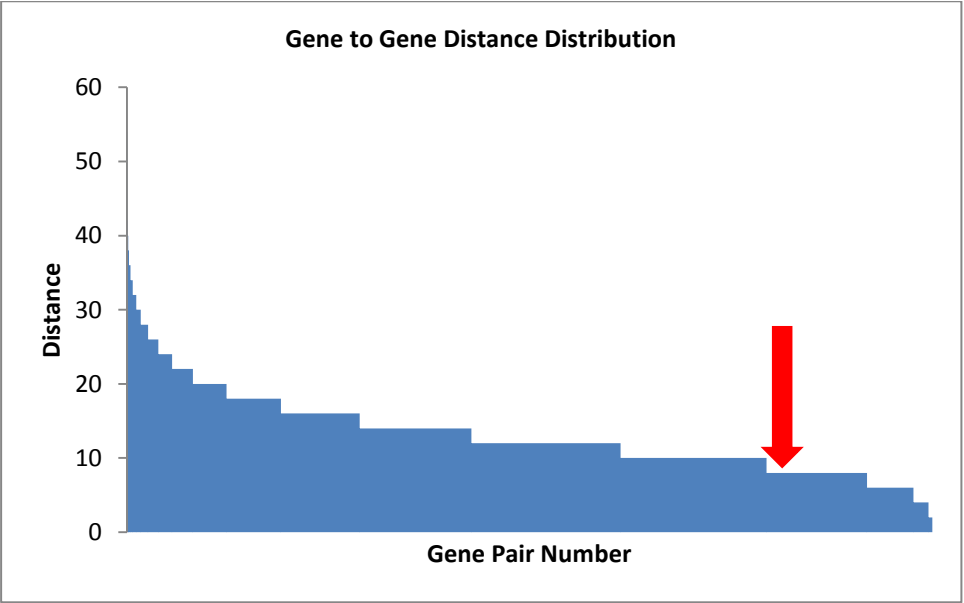

Supplement: Additional file 2: Figure S2 — Gene to Gene Distance Distribution. [file 1471-2105-13-197-S2.pdf]

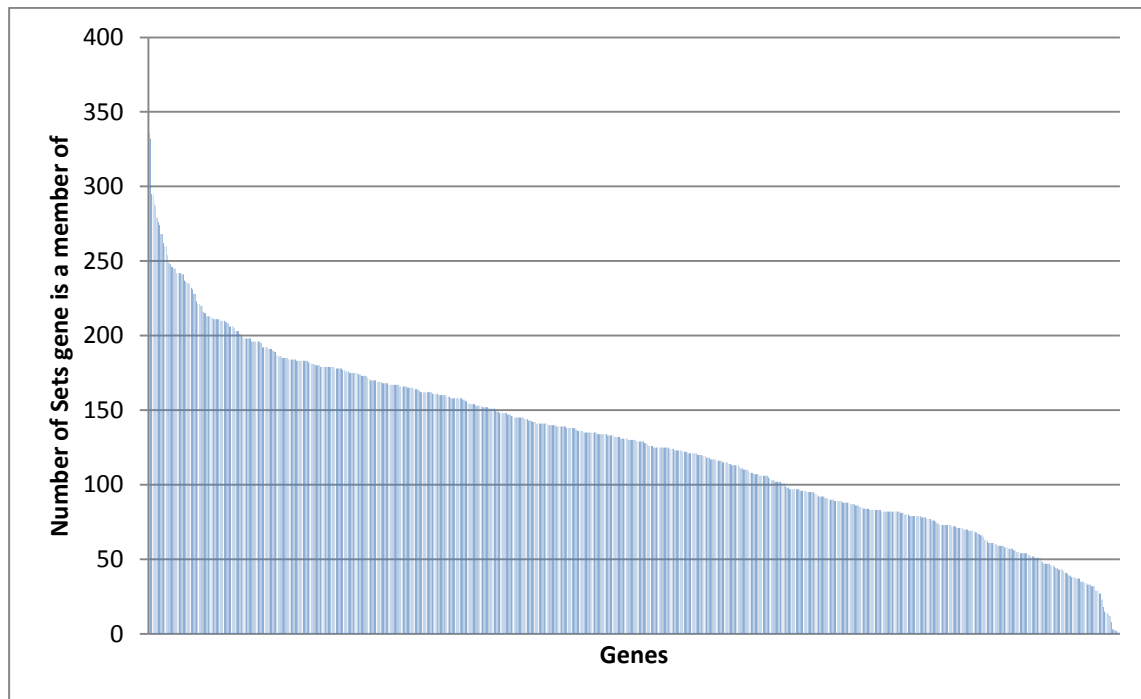

Supplement: Additional file 3: Figure S3 — Rank order distribution of the number of sets that each gene is contained in. [file 1471-2105-13-197-S3.pdf]

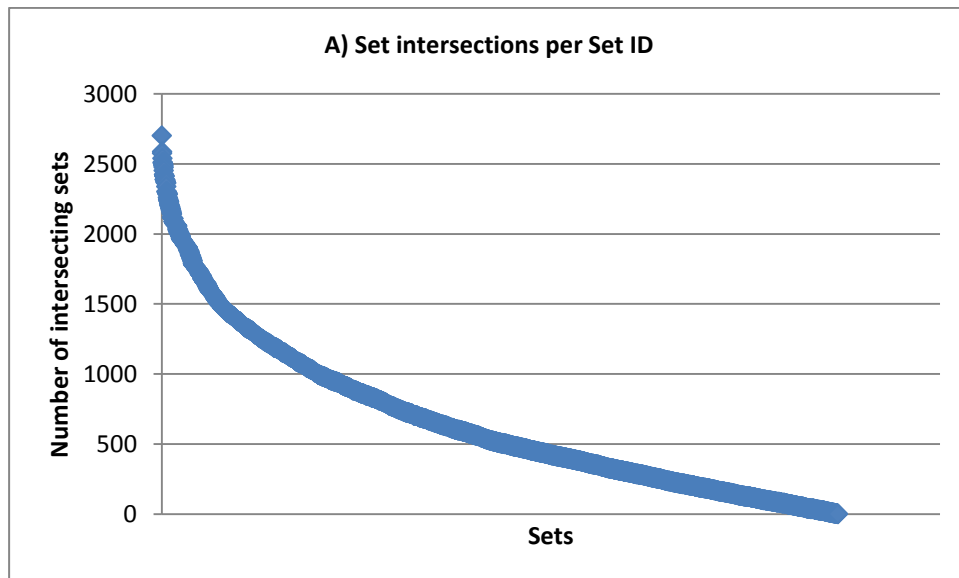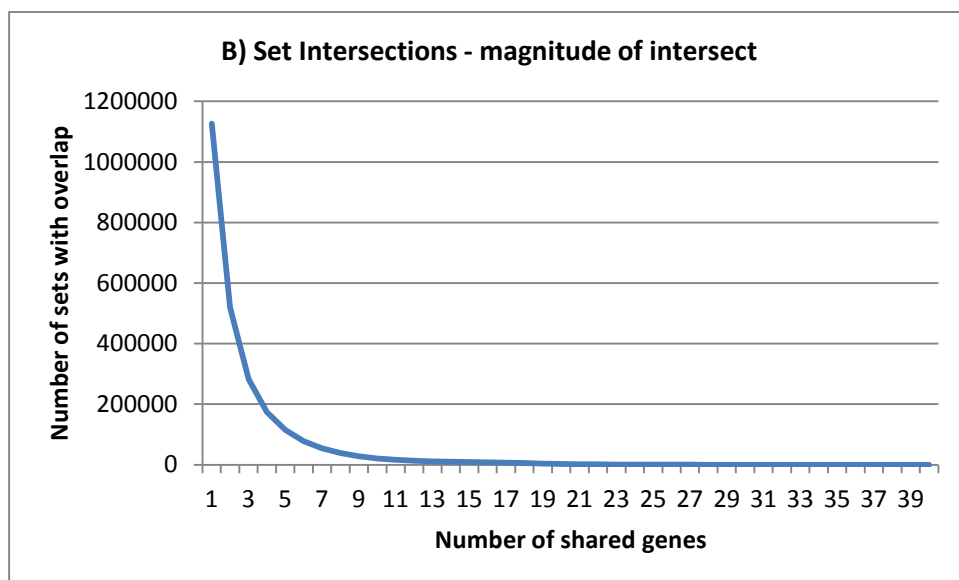

Supplement: Additional file 4: Figure S4 — A) number of other gene sets that intersect with each gene set. B) Number of occurrences of gene overlaps between sets as found by set intersections. [file 1471-2105-13-197-S4.pdf]

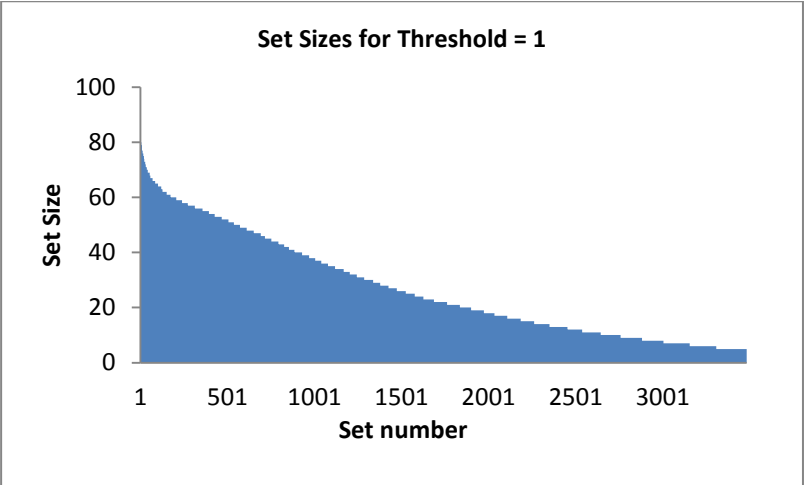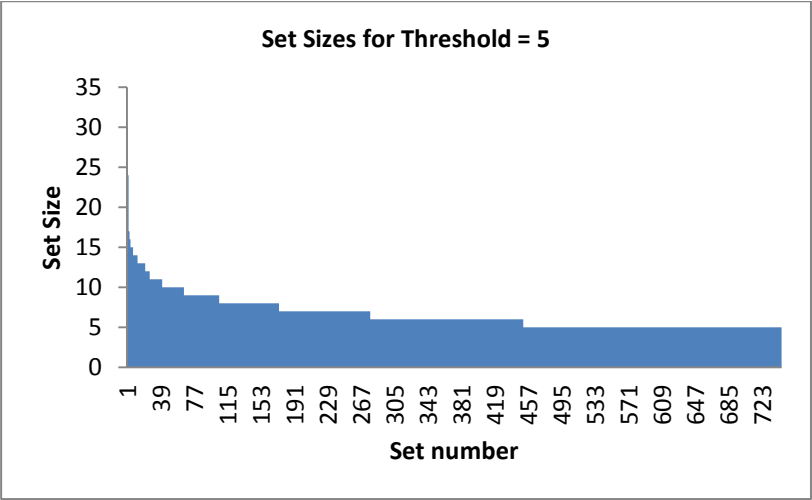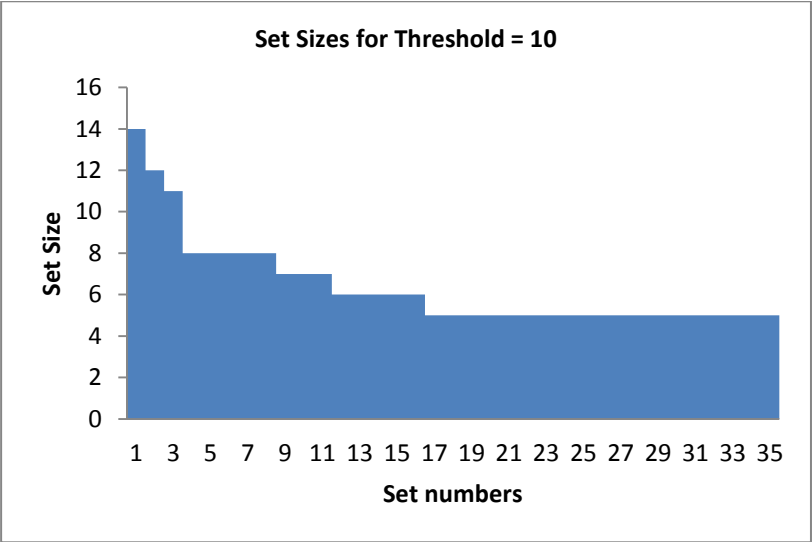

Supplement: Additional file 5: Figure S5 — Set sizes generated at each positive and negative arm of each principal component at thresholds 1, 5 and 10. [file 1471-2105-13-197-S5.pdf]

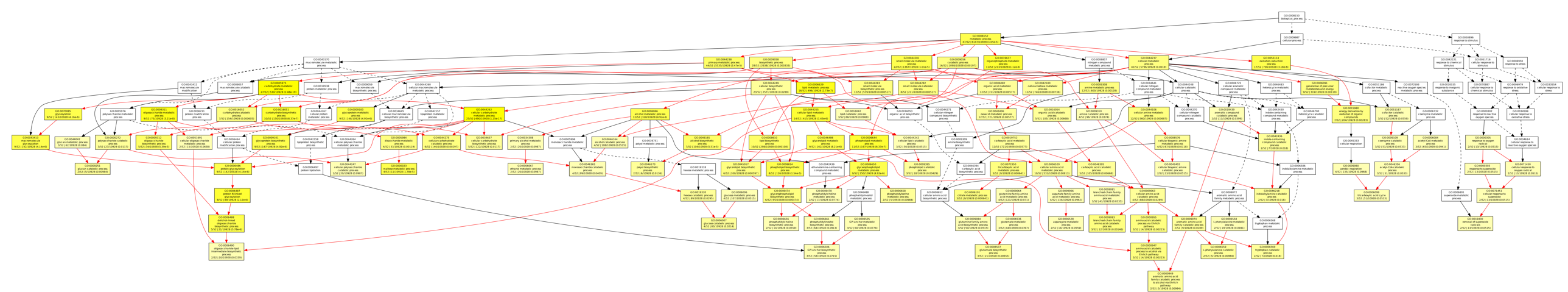

Supplement: Additional file 9 — GO Enrichment (Cellular Location) of the False Negatives from traditional GSA pathway sets. [file 1471-2105-13-197-S9.pdf]

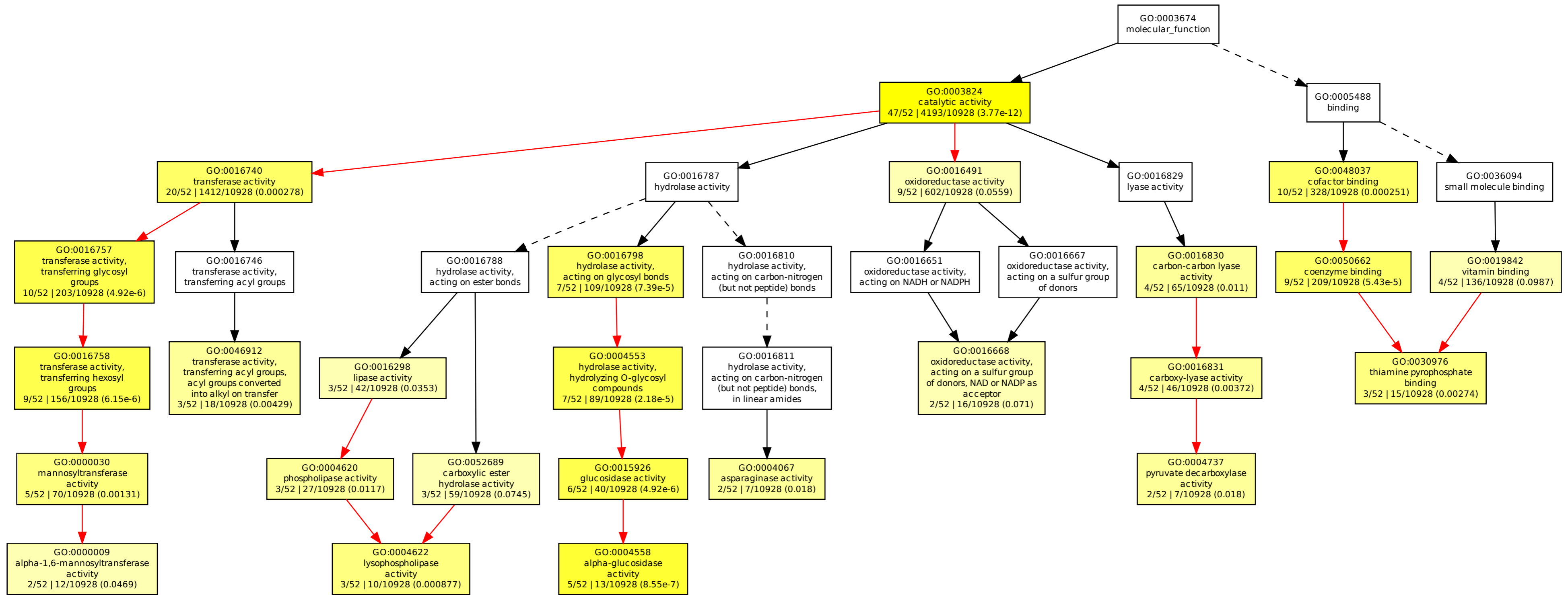

Supplement: Additional file 10 — Gene ID, Name and Description of metabolic genes found to be differentially expressed by GSA-PCA. [file 1471-2105-13-197-S10.pdf]

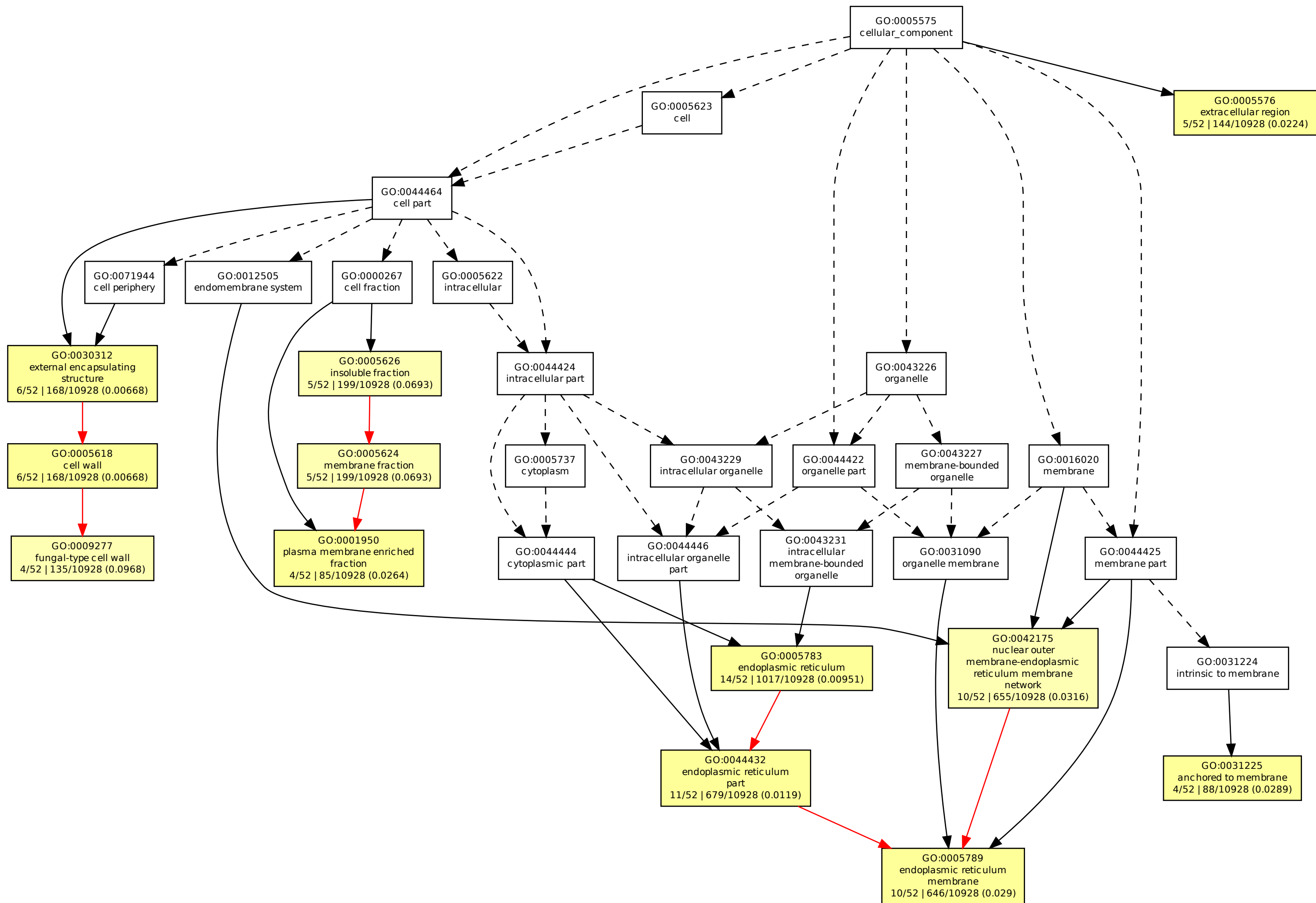

Supplement: Additional file 11 — The Gene Sets Generated with PCA Score Threshold 1. [file 1471-2105-13-197-S11.pdf]
